# Effectiveness of Mesoporous Silica Nanoparticles Functionalized with Benzoyl Chloride in pH-Responsive Anticorrosion Polymer Coatings

**DOI:** 10.1021/acsapm.3c00585

**Published:** 2023-07-12

**Authors:** Federico Olivieri, Fabio Scherillo, Rachele Castaldo, Mariacristina Cocca, Antonino Squillace, Gennaro Gentile, Marino Lavorgna

**Affiliations:** † Institute of Polymers Composites and Biomaterials, National Research Council of Italy, Via Campi Flegrei, 34, 80078 Pozzuoli, NA, Italy; ‡ Department of Chemical, Materials and Industrial Production Engineering, University of Naples Federico II, P.le Tecchio 80, 80125 Naples, Italy; § Institute of Polymers Composites and Biomaterials, National Research Council of Italy, P. le Enrico Fermi 1, 80055 Portici, NA, Italy

**Keywords:** mesoporous materials, nanoparticles, coatings, corrosion, smart release

## Abstract

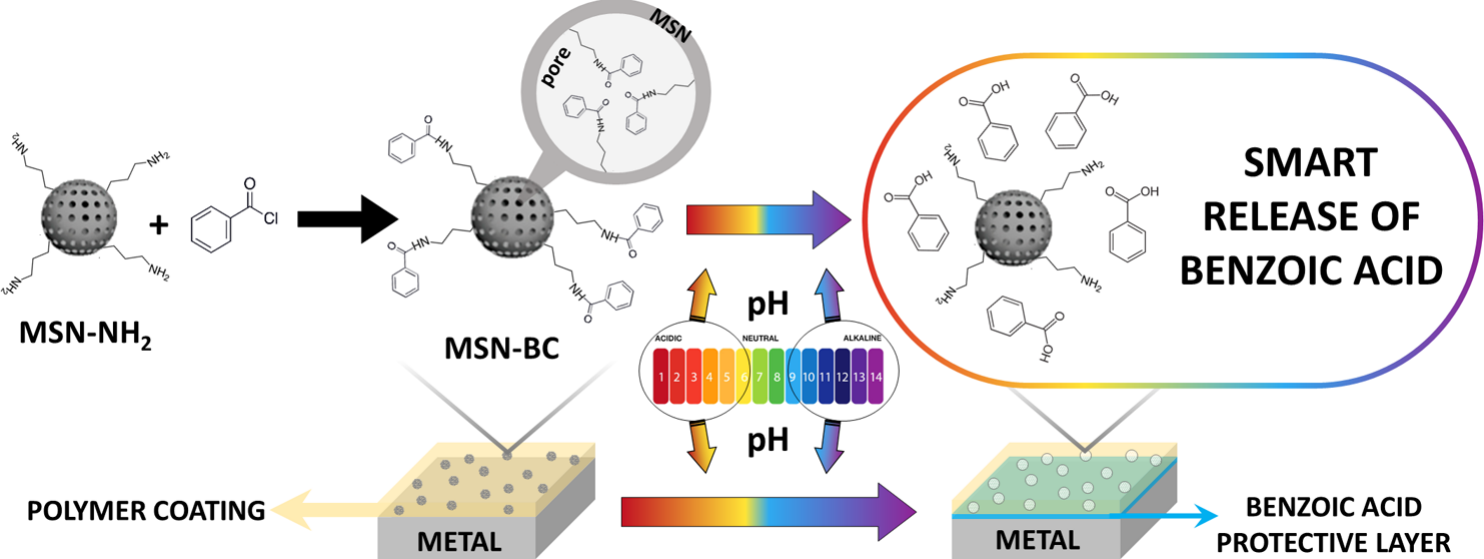

Smart polymer coatings embedding stimuli-responsive corrosion inhibitor nanocarriers are commonly exploited, in the literature, for the development of high-performance active coatings. In this work, high-surface-area amino-functionalized mesoporous silica nanoparticles (MSN-NH_2_) were developed with a one-step synthesis process and then functionalized with benzoyl chloride (MSN-BC) through a reaction with amino groups. MSN-BC are able to release benzoic acid (BA) in acid and alkaline conditions as a result of the hydrolysis of the pH-sensitive amide bond. MSN-BC were embedded in polymer coatings to exploit the pH-dependent release of corrosion-inhibiting BA. After an in-depth characterization of the developed functional nanoparticles and of their pH-dependent release kinetics of BA, MSN-BC were embedded in an acrylic matrix, realizing coatings for the corrosion protection of aluminum AA2024 alloys. Results demonstrate the effectiveness of the nanoparticles’ porous structure for a high loading of the anticorrosive active agent BA and the long-lasting efficiency of the coating for the corrosion protection of aluminum alloys, as validated by morphological and electrochemical impedance spectroscopy (EIS) measurements. EIS experiments were carried out with up to 21 days of exposure to a corrosive environment, revealing the potentialities of the acrylic coatings embedding MSN-BC for the protection of aluminum alloys.

## Introduction

Aluminum alloy AA2024 is a widely exploited material for its high mechanical properties and lightness, in particular in the aeronautical, aerospace, and construction fields.^1,2^ However, the presence of ligands, such as copper, magnesium, zinc, silicon, and, above all, precipitates they form, dramatically affect its corrosion resistance.^3^ For this reason, hexavalent chromium-based coatings were largely applied onto aluminum surfaces in industrial applications.^4^ However, the carcinogenicity of chromium-based materials currently limits their use as protective layers. Among the proposed alternatives, researchers developed rare-earth additives,^5^ tin oxide films,^6^ graphene-based coatings applied by chemical vapor deposition,^7^ and hybrid silica–zirconia-based coatings.^8^ Polymer coatings are also largely investigated to protect metal substrates, thanks to their easy applicability, low cost, and high barrier properties.^9−11^ Polymers can also be easily functionalized, realizing composite coatings, which act as physical barriers for metal protection, modulating the interaction with the substrate^12^ or tuning properties such as hydrophobicity.^13,14^

Generally, the most common problems of protective coatings involve their hydrolytic stability and the adhesion of the coating to the oxide layer naturally present on aluminum substrates, inducing the adsorption of water and ions at the interface and promoting the creation of a corrosive environment.^15^ Nevertheless, a good interaction between the coating and the substrate is not sufficient to ensure long-lasting protection from corrosion of aluminum substrates. In fact, once the passive barrier of the protective coating and the oxide layer is deactivated, corrosion processes irreversibly start.

In this context, the development of polymer-based smart coatings can ensure higher durability and protection effectiveness with respect to passive barriers.^16^ Indeed, the protective efficiency of polymer coatings can be improved by embedding smart systems containing corrosion inhibitors, which can be released on demand.^17,18^ That is, properly designed nanoparticles, such as silica nanoparticles and nanocapsules, are used as nanocarriers of corrosion inhibitors or self-healing agents, which are unloaded or released under appropriate external stimuli. In fact, nanocarriers have the advantage of being able to load high amounts of inhibitors, also shielding them by UV-induced deactivation and modulating their release.^19−23^

Among the most employed nanocarriers, mesoporous silica nanoparticles (MSN) are widely used in corrosion protection applications, thanks to their chemical stability, easy preparation, high surface area, pore volume, and, therefore, high loading capability.^16^ Indeed, MSN porosity allows the loading of a high quantity of inhibitors. Moreover, by localizing the corrosion inhibitors inside the nanoparticles’ porosity, difficulties connected with the dispersion of large amounts of corrosion inhibitors in polymer coatings are avoided.^24^ MSN have revealed their suitability for loading a wide range of corrosion inhibitors, such as benzotriazole (BTA),^24−26^ mercaptobenzothiazole (MBT),^27^ hydroxyquinoline (HQ),^28^ and sodium molybdate.^29^ Furthermore, MSN are easily functionalizable, in order to enhance/improve their releasing properties.^16^ However, MSN functionalization is usually subsequent to their synthesis and, in general, may involve more steps, limiting the reaction yield and the functionalization extent.^18^

Several works report the use of smart coatings containing MSN loaded with corrosion inhibitors, released through external triggers associated to corrosion processes, such as pH variation, redox reactions, and electrical potential.^16^ The triggered release mechanism may be activated by different smart capping systems based on a chemical or physical phenomenon. For instance, Keyvani et al.^30^ reported an effective system based on MSN functionalized with an amine/iron chloride coordination system that is able to block and release the corrosion-inhibiting molybdate anions, in response of pH changes. Keyvani et al. produced a protective coating based on epoxy resin embedding 1 wt % functionalized MSN loaded with sodium molybdate. This coating, tested by electrochemical impedance spectroscopy (EIS), showed higher corrosion resistance and exhibited a more intact surface in comparison with a coating embedding plain MSN for up to 8 weeks.^30^ With another approach, researchers have tailored the desorption of corrosion inhibitors from the porosity of MSN. For example, by loading corrosion inhibitors into MSN and blocking its undesired leaking with pH-dependent supramolecular switches,^31^ or by developing blocking systems involving the corrosion inhibitor itself, such as BTA–metal ion complexes, which are stable in neutral conditions and become more soluble by increasing the pH variation from neutrality.^24,25^ In the first case, self-assembled nanophase particle (SNAP) coatings containing stimuli-responsive MSN were realized. These coatings were able to extend the lifetime of the underlying aluminum alloy by hosting the anticorrosive agent and releasing it under acid and alkaline stimuli, simultaneously suppressing corrosion activities on microanodic and microcathodic regions.^31^ In the latter, MSN were loaded with benzotriazole (BTA) and capped with different metal cations forming pH-responsive complexes and the nanoparticles were embedded in polymer coatings for the protection of metal substrates. These coatings were able to preserve from corrosion the underlying metals in both acid and alkaline environments, with significant improvement with respect to the corresponding coating containing a freely dispersed anticorrosive agent, being able to preserve BTA from photodegradation and release the anticorrosive agent when needed. Hollamby et al.^12^ also realized protective polymer coatings containing MSN loaded with BTA, which outperformed the corresponding polymer coating containing freely dispersed BTA. Indeed, the amounts of freely dispersed corrosion inhibitors in the polymer coating needed to be 10-fold greater to obtain similar protection. Additionally, MSN have a minimal effect on the coating barrier and adhesion properties while enabling active corrosion protection.

As it appears, the development of stimuli-responsive release systems for corrosion inhibitors loaded or grafted onto MSN is very appealing. Nevertheless, the proper design of smart release systems may involve several steps from the synthesis of MSN, their functionalization and purification, to the loading and stopping of the corrosion inhibitor, therefore resulting in a quite complex and/or expensive process.^16^

Here, we present a facile, high-throughput synthesis process with few steps for a corrosion inhibitor nanosystem based on MSN, its application in a commercial polymer formulation used for coating an aluminum alloy substrate, and the analysis of its protective efficiency. In particular, two reaction steps and an intermediate etching step are needed to obtain stimuli-responsive corrosion inhibitor nanocarriers: amino-functionalized MSN were obtained through a one-step and high-yield synthesis process, mildly etched and reacted with benzoyl chloride (BC) through a nucleophilic addition/elimination mechanism. From the dissociation of the pH-sensitive amide bond, the corrosion inhibitor benzoic acid (BA)^32^ is released. The release kinetics of BA from the nanoparticles was evaluated through simulated release tests in acid and basic conditions. The as-obtained nanoparticles were embedded into commercial acrylic coatings deposited onto the AA2024 alloy, and their anticorrosion efficiency was assessed through electrochemical impedance spectroscopy (EIS) measurements.

## Experimental Section

### Materials

Tetraethyl orthosilicate (TEOS), 3-aminopropyltriethoxysilane (APTES), hexadecyltrimethylammonium chloride (CTAB), triethanolamine (TEAH_3_), benzoyl chloride (BC), triethylamine (TEA), hydrochloric acid (HCl, assay 36%), sodium hydroxide (NaOH), anhydrous tetrahydrofuran (THF), sodium chloride (NaCl), ethanol, and acetone were purchased from Sigma-Aldrich. An acrylic resin, named Paraloid B72 (methyl acrylate/ethyl methacrylate), was obtained from Antichità Belsito (Rome, Italy). Aluminum alloy AA2024 under T3 temper with the following composition (atomic %): Al 90.7–94.7, Cu 3.8–4.9, Mg 1.2–1.8, Mn 0.3–0.9, other elements <0.5 each one, as defined by Aerospace Specifications Metals (ASM) Inc, was purchased from Pechiney.

### Synthesis of MSN-NH_2_ and Grafting with BC

Amino-modified mesoporous silica nanoparticles (MSN-NH_2_) were prepared through a modified high-yield procedure.^33^ CTAB was employed as a templating agent. CTAB, TEAH_3_, and H_2_O were mixed in a 0.06:8:80 molar ratio for 1 h at 80 °C (pH ≈ 10). Then, TEOS and APTES in a weight ratio of 9:1 were added in the following sequence: TEOS was added first to induce its prehydrolysis and APTES was added after 10 min. At this point, the synthesis was continued for 1.5 h. MSN-NH_2_ were collected through filtration and washed with a 1.5 M ethanol HCl solution. Then, MSN-NH_2_ were washed with bidistilled water until neutral and dried under vacuum overnight. For yield calculation, taking into account the molar ratio of TEOS and APTES (9:1) and approximating the elemental composition of the final MSN-NH_2_ to (9/10) SiO_2_ * (1/10) (SiO_3/2_CH_2_CH_2_CH_2_NH_2_) (molecular weight 65.0939 g/mol, omitting in this way the contribution of terminal hydroxyl groups), the maximum product/reagent weight ratio that could be obtained by the reaction (P/R_MAX_) was found to be 0.3029, due to eq 1
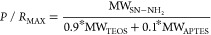
1

Therefore, the yield of the reaction was found by eq 2
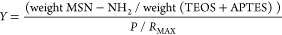
2Before reaction with BC, MSN-NH_2_ were surface-etched with NaOH. In detail, MSN-NH_2_ were treated at room temperature with 1 mM NaOH for 24 h. Then, the obtained product was filtrated, washed with bidistilled water until neutral, and dried in an oven under vacuum. The obtained particles were coded MSN-NH_2_-E.

Finally, MSN-NH_2_-E were reacted with BC as follows: 0.4 g of MSN-NH_2_-E, 1 mL of BC, 0.4 mL of TEA, and 10 mL of THF were added to a round-bottom flask, and the mixture was stirred at room temperature for 20 h under a nitrogen atmosphere.^29^ The obtained product was collected through filtration, washed with ethanol, and dried overnight in a vacuum oven. The obtained nanoparticles were coded MSN-BC.

For comparison, nonfunctionalized MSN were synthesized with the same procedure, using 10 g of TEOS and without the addition of APTES. Furthermore, to evaluate the advantage of the single-step synthesis of MSN-NH_2_, amino-functionalized MSN were also realized through a two-step synthesis process using the same TEOS/APTES weight ratio employed for MSN-NH_2_. On the basis of the maximum yield evaluated by eq 2 for plain MSN, 0.2596 g of MSN were reacted with 0.1 g of APTES in this way: MSN were dispersed in 10 mL of ethanol, under stirring, and then APTES was added and the reaction was stirred for 1.5 h. The obtained nanoparticles, coded MSN-NH_2_-2, were washed with distilled water and collected by filtration.

### Coating Preparation

A 10 wt % acetone solution of Paraloid B72 was prepared. Then, MSN-BC (5 wt % with respect to Paraloid B72) were added to the solution and ultrasonicated with a Sonics Vibracell ultrasonic processor (Newton, 500 W, 20 kHz), at 25% of amplitude for 10 min, with 15/15 s on/off cycles; to avoid overheating, the dispersion was kept in an ice–water bath during the treatment. This mixture was poured onto aluminum substrate samples in order to obtain coatings 30 ± 4 μm thick by film casting. These samples were coded ACR-MSN. For comparison, another set of aluminum substrates was coated with a Paraloid B72 solution containing BA in the same molar amount of the total BC grafted in MSN-BC employed for the ACR-MSN coating, namely, 0.45 wt % with respect to Paraloid B72. This set of samples was coded ACR-BA. Then, coatings of Paraloid B72 without additional components were applied onto a set of aluminum substrates, and these samples were coded ACR. ACR, ACR-BA, and ACR-MSN coatings were also realized on glass substrates, following the same procedure above described.

### Characterization

Morphological analysis of MSN-NH_2_, MSN-NH_2_-E, and MSN-BC was performed through transmission electron microscopy (TEM) using an FEI Tecnai G12 Spirit Twin (LaB_6_ source) at 120 kV acceleration voltage (FEI, Eindhoven, the Netherlands). TEM images were collected on an FEI Eagle 4 k CCD camera. Before the analysis, nanoparticles were dispersed in ethanol through sonication using a Sonics Vibracell (Newtown, CT) ultrasonic processor (500 W, 20 kHz) at 25% of amplitude for 5 min and then collected on carbon-coated copper grids.

Textural properties of MSN-NH_2_, MSN-NH_2_-E, and MSN-BC were analyzed by N_2_ adsorption at 77 K using a Micromeritics ASAP 2020 analyzer (Norcross, Georgia). The specific surface area of the nanoparticles was determined from the linear part of the Brunauer–Emmett–Teller (BET) equation. Nonlocal density functional theory (NLDFT) was applied to the nitrogen adsorption isotherms to evaluate the pore size distribution of the nanoparticles. Prior to the analyses, all samples were degassed at 100 °C under vacuum (P <10–5 mbar) and all of the adsorption measurements were performed using high-purity gases (>99.999%).

Thermogravimetric analysis (TGA) was conducted on a PerkinElmer Pyris Diamond TG/DTA (Waltham, MA). TGA was performed on MSN-NH_2_, MSN-NH_2_-E, MSN-BC, MSN, and MSN-NH_2_-2 by a 10 min isothermal scan at 100 °C and then heating up to 900 °C with a heating rate of 20 °C/min in an oxidative atmosphere.

Energy-dispersive X-ray analysis (EDX) was used to evaluate the functionalization extent of MSN-NH_2_-E. The analysis was performed by means of an FEI Quanta 200 FEG SEM equipped with an Oxford Inca Energy System 250 and an Inca-X-act LN2-free analytical silicon drift detector. Before the analysis, a thin layer of MSN-NH_2_-E was deposited on an aluminum stub. Then, the sample was analyzed at 30 kV acceleration voltage in low vacuum mode.

Fourier transform infrared (FTIR) spectroscopy in attenuated total reflectance (ATR) mode was performed by a PerkinElmer Spectrum One FTIR spectrometer (Waltham, MA), equipped with an ATR module, using a resolution of 4 cm^–1^ and 32 scan collections on MSN-NH_2_, MSN-NH_2_-E, and MSN-BC.

Benzoic acid release in water was analyzed by ultraviolet (UV) spectrophotometry, performed with a Jasco V570 UV spectrophotometer (Jasco, Easton, MD). 0.03 mg/mL of MSN-BC were dispersed in water at pH 7, in a HCl–water solution at pH 1.5, and in a NaOH–water solution at pH 12.5 at 25 °C. BA release was also monitored by the immersion of ACR, ACR-BA, and ACR-MSN coatings on glass in a HCl–water solution at pH 1.5. The kinetics of BA release was monitored through the UV spectra of the supernatant solution, using pre-recorded BA calibration curves (Figure S1). Measurements were performed in triplicate. Average values and standard deviation values were calculated.

EIS was performed with a Solartron 1280 onto three different aluminum AA2024 substrates coated with ACR, ACR-BA, and ACR-MSN separately. The samples were exposed to a NaCl 3.5 wt % aqueous solution for 3 weeks and the electrochemical properties were evaluated immediately (time 0) after 1 day, 1 week, and 3 weeks. Tests were conducted with a two-electrode cell setup (the coated aluminum samples as the working electrode and platinum as the counter electrode), an amplitude sinusoidal voltage of 50 mV, and a frequency range of 10^–1^–10^4^ Hz on a 30 mm diameter circular area.

Morphological analysis of ACR-, ACR-BA-, and ACR-MSN-coated aluminum AA2024 substrates before and after exposure to NaCl solution was investigated by a Lynx EVO stereomicroscope (Vision Engineering Ltd, Milan, Italy) and by scanning electron microscopy (SEM) analysis, using an FEI Quanta 200 FEG SEM (FEI, Eindhoven, the Netherlands) in high vacuum mode.

## Results and Discussion

**Figure 1:**
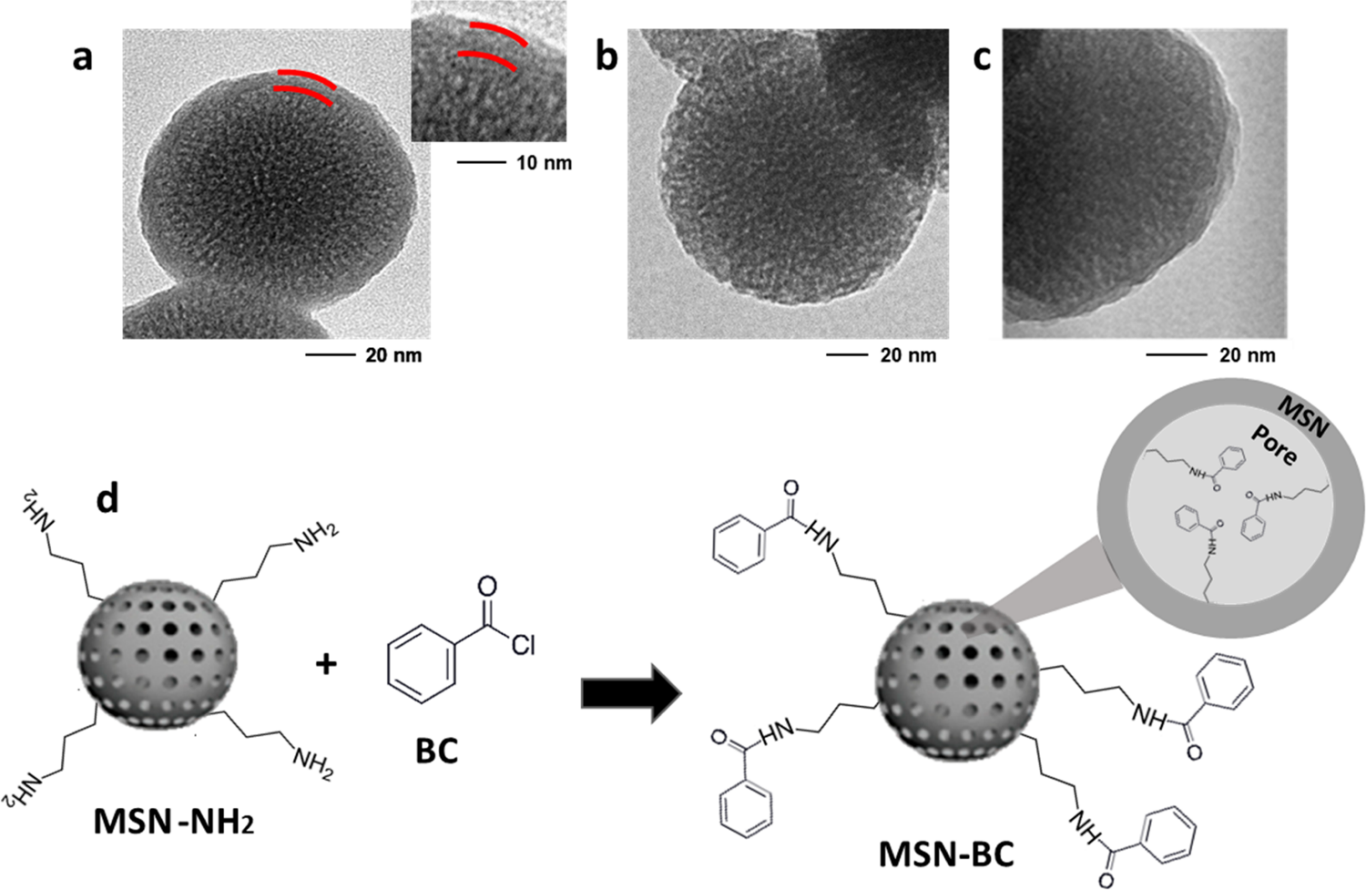
TEM images of MSN-NH_2_ (a), MSN-NH_2_-E (b), and MSN-BC (c). Scheme of BC grafting on MSN-NH_2_ (d).

In order to synthesize functional mesoporous silica nanoparticles able to release under appropriate stimuli a sustainable anticorrosive agent, namely, benzoic acid, a one-step high-yield/high-throughput synthetic protocol was developed to produce amino-functionalized MSN in the presence of an organic pore templating agent, CTAB, combined with a suitable amine (TEAH_3_). With this approach, MSN-NH_2_ are obtained through a one-step reaction with a yield of about 92%, as calculated by eq 2. The obtained MSN-NH_2_ have an average diameter of 106 ± 28 nm and a quite homogeneous wormlike porosity, as shown by the TEM image in Figure 1a. At higher magnification, MSN-NH_2_ show a thin external shell characterized by higher density, evidenced in the inset.

**Figure 2:**
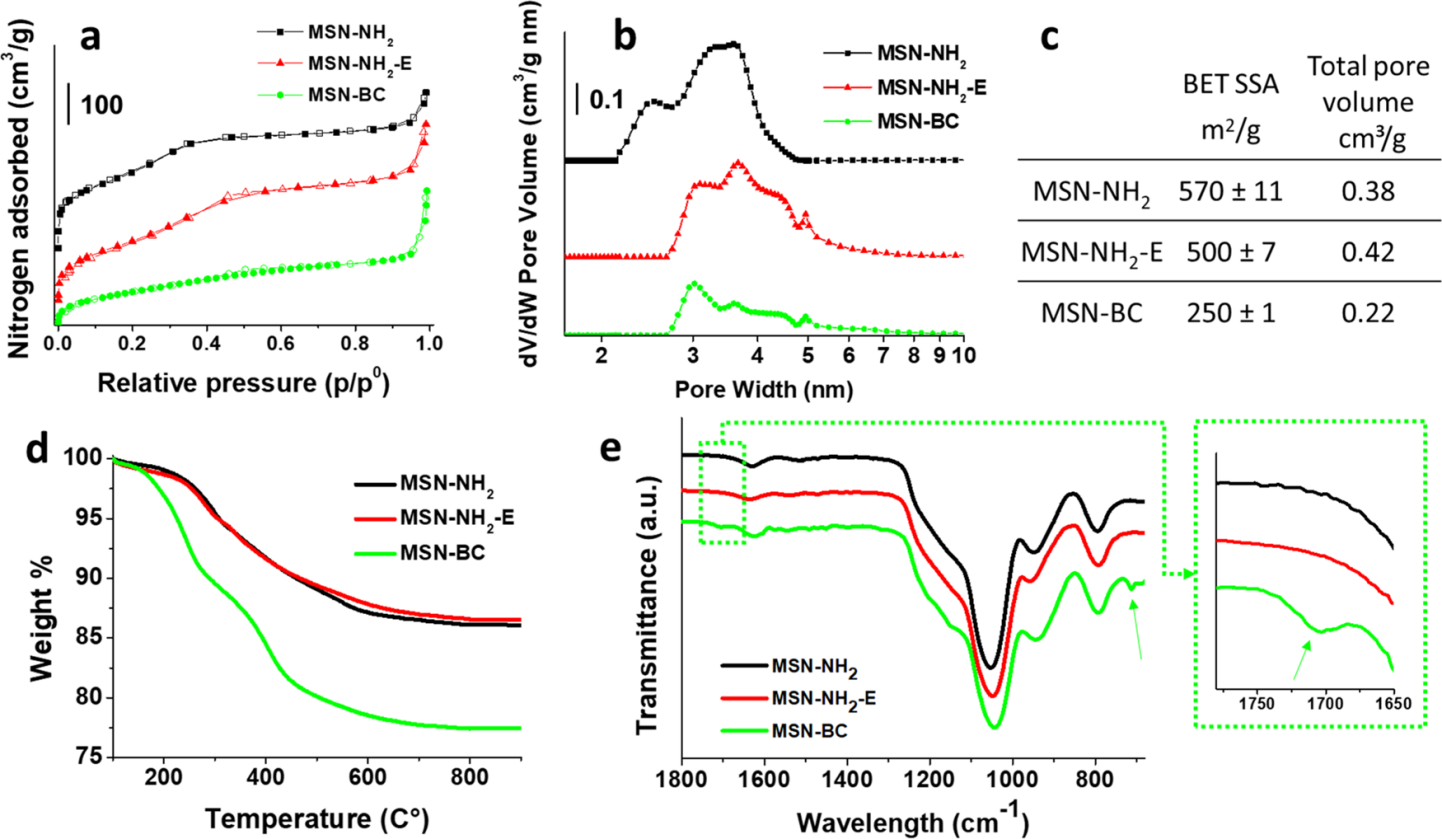
Nitrogen adsorption/desorption isotherms (a), NLDFT pore size distribution (b), values of BET SSA and pore volume (c), TGA traces (d), and FTIR spectra (e) of MSN-NH_2_, MSN-NH_2_-E, and MSN-BC.

Nitrogen adsorption analysis of MSN-NH_2_ (Figure 2a,b) shows a dual porosity distribution with a major peak of porosity centered around 3.5 nm and a minor peak centered around 2.5 nm. MSN-NH_2_ are characterized by a BET SSA of 570 m^2^/g and a specific pore volume of 0.38 cm^3^/g, which are comparable to results obtained for plain MSN, reported in Figure S2 (a BET SSA of 620 m^2^/g and a pore volume of 0.39 cm^3^/g). Despite the porosity of MSN-NH_2_ being accessible to gas adsorption, the amount of grafted benzoyl groups on MSN-NH_2_ after the reaction with benzoyl chloride resulted in a very low yield (< 2%, comparable to the grafting yield obtained on nonporous silica nanoparticles^18^), possibly because the external dense layer (Figure 1a) precludes the diffusion of the BC molecules toward the inner part of the nanoparticles.

In order to remove such a dense external layer, mild etching of the MSN-NH_2_ nanoparticles was carried out, by treatment at room temperature with an aqueous 1 mM NaOH solution for 24 h, obtaining the etched amino-functionalized nanoparticles MSN-NH_2_-E. This mild etching procedure did not modify the FTIR spectrum of MSN-NH_2_, which was identical to that of MSN-NH_2_-E, and did not induce an appreciable weight loss, thus suggesting that it occurred without a significant change in the nanoparticle composition. Nevertheless, as shown by the TEM image in Figure 1b, the etching effectively removes the dense layer evidenced for MSN-NH_2_. Moreover, nitrogen adsorption analysis of MSN-NH_2_-E shows a different pore size distribution ranging from 3 to 5 nm (Figure 2b). MSN-NH_2_-E also show a slightly higher pore volume of 0.42 cm^3^/g and a decreased surface area of 500 m^2^/g with respect to MSN-NH_2_ (Figure 2c), compatible with the presence of relatively larger pores and the disappearance of the smaller pore fraction.

TGA curves of MSN-NH_2_-E and MSN-NH_2_ (Figure 2d) show a comparable residual weight at 900 °C, confirming the absence of an appreciable effect of the etching on the composition of the nanoparticles. TGA was also used to perform a preliminary quantitative evaluation of amino groups grafted onto MSN. Comparing the TGA curve of MSN-NH_2_-E to the TGA curve of nonfunctionalized MSN (Figure S3), which present weight residual values at 900 °C of 86.5 ± 0.9 and 97.5 ± 0.4 wt %, respectively, the amount of amino groups effectively grafted on the porous surface of MSN can be calculated by approximating this weight loss difference to the degradation and release of the aminopropyl moieties. With this approximation, this weight loss difference, about 11 wt %, corresponds to 1.89 × 10^–3^ mol of amino groups per gram of MSN-NH_2_-E.

The quantitative evaluation of amino groups in MSN-NH_2_-E was confirmed by EDX analysis, evaluating the weight ratio between carbon and silicon. These elements were chosen because nitrogen and oxygen EDX peaks were convoluted (Figure S4) and their quantitative evaluation was affected by a high error. The C/Si weight ratio was 0.146 ± 0.010. As the amount of carbon is strictly related to the amount of aminopropyl moieties, by stoichiometric considerations, the C/Si weight ratio corresponds to (1.74 ± 0.10) × 10^–3^ mol of amino groups per gram of MSN-NH_2_-E. This result is in good agreement with TGA results and well compatible with the used reagent ratio, as the final composition of MSN-NH_2_-E corresponds to a TEOS/APTES weight ratio of about 9:1.2.

Considering the BET surface area of MSN-NH_2_-E, the amount of aminopropyl groups per surface unit can be calculated as 3.47 × 10^–6^ mol/m^2^, more than 17-fold higher with respect to the functionalization extent achieved on nonporous silica nanoparticles with similar average size (in the range of 0.100 × 10^–3^ mol/g) and corresponding to about 52% of the full estimated monolayer coverage of the MSN surface with APTES (about 4 groups/nm^2^, corresponding to 6.64 × 10^–6^ mol/m^2^).^34^

Then, the etched amino-functionalized nanoparticles were reacted with benzoyl chloride (BC) through a nucleophilic addition/elimination mechanism, allowing the grafting of benzoic groups onto MSN pore surfaces through amide bonds (see Scheme in Figure 1d). The reaction of BC with amino groups is an effective derivatization of amines, previously reported in the literature,^35^ also for the modification of nanosilica.^36^ The obtained nanoparticles MSN-BC still show a wormlike porosity, as evidenced in the TEM image in Figure 1c. Moreover, they also show an external layer characterized by different contrast, whose thickness ranges between 2 and 7 nm. As a consequence of grafting, MSN-BC surface area and porosity are significantly reduced with respect to MSN-NH_2_-E: the specific surface area and pore volume of MSN-BC are 250 m^2^/g and 0.22 cm^3^/g, respectively (Figure 2c). In particular, larger porosity is reduced upon functionalization, as shown by the MSN-BC pore size distribution (Figure 2b), indicating a possibly large amount of covalently loaded benzoic groups.

The occurrence of the reaction between BC and the MSN-NH_2_-E amino groups was confirmed by FTIR analysis, which evidenced the appearance of a stretching carbonyl band centered at 1705 cm^–1^, typical of amide groups, and a band centered at 711 cm^–1^, typical of the aromatic C–H bending (Figure 2e).^37^ Due to the benzoylation of the aminosilane, the position of the carbonyl band in MSN-BC was significantly different from that shown by both the BC precursor as well as from BA (Figure S5). Moreover, a significantly higher thermo-oxidative degradation characterizes the BC-grafted nanoparticles in comparison to the pristine nanoparticles. Indeed, MSN-BC (Figure 2d) shows thermo-oxidative degradation starting at about 120 °C, with two degradation steps centered at about 250 and 400 °C, leading to a residual weight of about 77.0 ± 1.0% at 900 °C. Comparing MSN-BC and MSN-NH_2_-E residual weights, the amount of BC grafted is estimated at about 8.5 ± 1.0 wt % (Figure 2d), more than 4 times higher than the amount of BC grafted onto nonporous silica nanoparticles.^18^ This corresponds to about 8.02 × 10^–4^ mol per gram of MSN-BC or, when expressed with respect to the precursor MSN-NH_2_-E, to 8.76 × 10^–4^ mol per gram of MSN-NH_2_-E. This result is very interesting because despite the high hindrance due to the porous structure of MSN and the progressive decrease of the accessibility of the inner pores during the reaction with BC, demonstrated by the low SSA of MSN-BC with respect to MSN-NH_2_-E, a high yield of the reaction of BC (about 50%) with the amino groups is achieved. Moreover, considering the weight loss of the two-step synthesized MSN-NH_2_-2 (Figure S6), which is about 4.7 wt % (corresponding to 8.09 × 10^–4^ mol of amino groups per gram of nanoparticles), the amount of grafted BC groups (8.76 × 10^–4^ mol per gram of MSN-NH_2_-E) exceeds the total amount of amino groups in MSN-NH_2_-2, confirming the convenience of exploiting the one-step synthesis of MSN-NH_2_.

**Figure 3:**
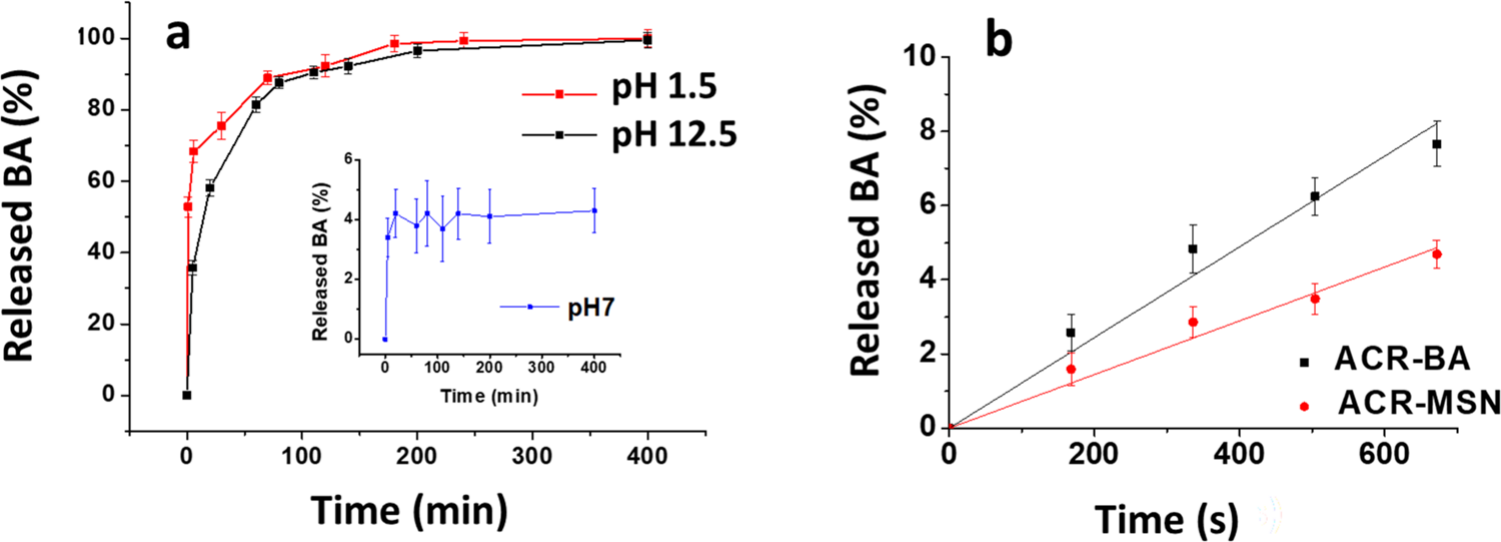
Benzoic acid release from MSN-BC at pH 1.5, 12.5, and 7.0 (a); benzoic acid release from ACR-BA and ACR-MSN at pH 1.5 (b).

Exploiting the susceptibility of the amide bonds to hydrolysis, with the designed release of benzoic acid,^38^ MSN-BC were analyzed by performing release tests in acid and basic conditions. The release kinetics of BA from MSN-BC at pH 1.5 and 12.5 is shown in Figure 3a: at pH 1.5, the release of BA is very fast, with 70% of BA being released in the first 10 min, while at pH 12.5, about the same amount of BA is released in about 50 min; then, the release of about 90% of the grafted BA is completed in around 70 min at pH 1.5 and 90 min at pH 12.5. At pH 7.0, a very low release of BA was observed during the same timescale.

To better evaluate the kinetics of BA release from the acrylic coating, ACR-BA and ACR-MSN coatings, immersed in acid solutions at pH 1.5, were monitored by UV spectroscopy. Results shown in Figure 3b revealed slow release in both cases (less than 8% of BA released after 1 month for ACR-BA and less than 5% for ACR-MSN), but with a different release rate, easily identifiable by the linear trend of the curves as their slopes (0.29% BA/day and 0.17% BA/day, respectively). Therefore, the additional dissociation of the MSN-BC amide bond, involved in the release of BA from ACR-MSN coatings subjected to acid stimuli, leads to a more delayed release of BA with respect to freely dispersed BA in ACR-BA. To consider eventual effects due to the acrylic matrix, the ACR coating was also exposed to the same aging environment, with no appreciable effects.

**Figure 4:**
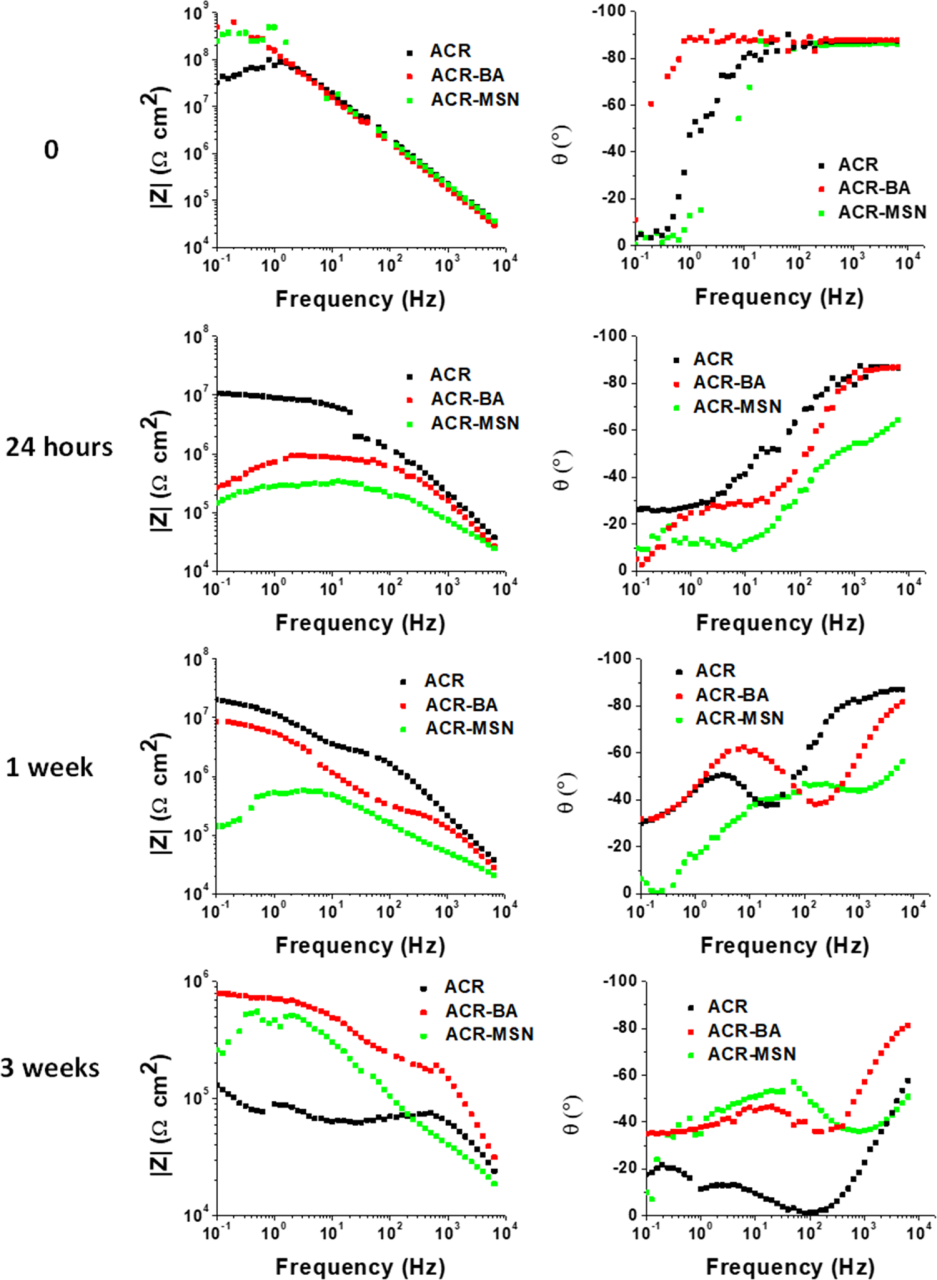
EIS Bode plots of ACR, ACR-BA, and ACR-MSN evaluated at time zero, after 24 h, 1 week, and 3 weeks of exposure.

EIS measurements on ACR-MSN, ACR-BA, and ACR, reported in Figure 4, show the evolution of the protective effect of the coating exposed to a 3.5 wt % NaCl solution. Despite the pH-dependent release properties of the synthesized MSN-BC, EIS experiments were performed at neutral pH, as it is well established for different systems that local pH changes, occurring during weathering of metal substrates in contact with neutral NaCl solutions, are able to promote the on-demand release of corrosion inhibitors from nanocarriers triggered at acid or basic pH values.^39^

The ACR coating showed the highest impedance curve after the very first period of exposure (24 h), probably due to the high homogeneity of the plain coating. Its protective effect considerably decreased after 1 week and irremediably decreased after 3 weeks when both ACR-BA and ACR-MSN coatings showed better protection than ACR. Indeed, in the case of plain ACR, once the corrosive solution penetrates the coating, it irreparably starts the corrosive process. Also, after 24 h, while ACR and ACR-MSN were only characterized by the high-frequency time constant, ACR-BA showed the insurgence of a low-frequency time constant, which indicated that the freely dispersed BA acted as a passive barrier onto the metal surface. Indeed, a high-frequency time constant is associated with the coating capacitance, and a low-frequency time constant is related to the resistance of the metal/oxide/coating interface.^40,41^ Then, after 1 week, a low-frequency time constant was also shown in ACR phase angle measurement, probably due to the formation of an aluminum oxide protective layer. Contextually, ACR-BA showed the best anticorrosive performance, revealing that freely dispersed BA may have created a passivating barrier on the metal surface.

The substantial contribution of MSN-BC is clearly evident after 3 weeks when its low-frequency time constant becomes predominant with respect to ACR-BA, demonstrating the highest long-lasting protection.

**Figure 5:**
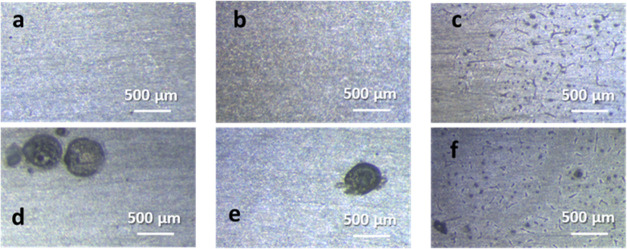
Optical images of ACR (a, d), ACR-BA (b, e), and ACR-MSN (c, f) before (a–c) and after (d–f) 3 weeks of NaCl exposure.

On the contrary, comparing the 1-week and 3-week phase angles of ACR-BA, it is evident that the freely dispersed benzoic acid progressively exhausts its protective action, probably because the corrosive solution has already overcome the passivating barrier created by BA. This result is in accordance with the release tests performed on ACR-BA in simulated acidic corrosive environments, shown in Figure 3b, which revealed a faster release of BA from the ACR-BA coating with respect to the ACR-MSN coating. Indeed, the amide bond dissociation involved in ACR-MSN stimuli-responsive release leads to a lower release rate of the anticorrosion agent, ensuring a longer-lasting efficiency. The above-mentioned results were confirmed by morphological analyses, performed by optical microscopy and SEM. Figure 5 shows the optical microscopy images of the coated aluminum substrates before NaCl solution exposure (Figure 5a–c) and after 3 weeks (Figure 5d–f). The embedding of MSN-BC into the acrylic coating induced the formation of wrinkles, as visible in Figure 5c,f, which are not associated with corrosion processes. After 3 weeks of exposure to NaCl, corrosion spots of about 500 μm diameter were observed on the surfaces of ACR (Figure 5d) and ACR-BA (Figure 5b), while corrosion pits were considerably smaller (about 100 μm wide) in ACR-MSN (Figure 5f).

**Figure 6:**
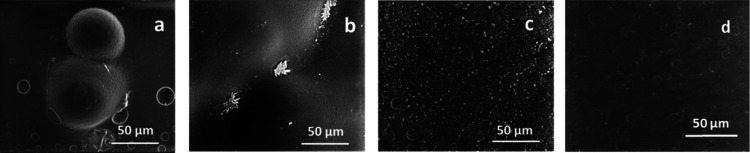
SEM analysis on ACR (a), ACR-BA (b), and ACR-MSN (c) samples after exposure to NaCl for 3 weeks. ACR-MSN after exposure to NaCl for 3 weeks and after washing with distilled water (d).

Moreover, SEM analysis of samples exposed to NaCl solutions for 3 weeks showed the presence of large bubbles on the polymer coating surface, either for ACR (Figure 6a) or for ACR-BA (Figure 6b), deriving from gas developed during corrosion phenomena. The ACR-MSN surface (Figure 6c), on the other hand, did not show evidence of corrosion phenomena, similar to that shown in SEM images of ACR and ACR-BA. For ACR-MSN, after 3 weeks of testing, only a diffuse presence of 1–2 μm particles on the sample surface was observed, ascribed to the formation of salt crystals on the sample surface, revealing the coating’s ability to accumulate the charges of the solution on its external surface. SEM analysis of ACR-MSN after washing with distilled water confirmed the salt crystal nature of the observed particles on the ACR-MSN surface, which were dissolved by the water treatment, leaving a still homogeneous and undamaged coating surface well visible (Figure 6d). Therefore, SEM analysis of the coated aluminum samples confirmed the major effectiveness of the BC-grafted nanoparticles in protecting the substrate from corrosion phenomena.

## Conclusions

In this work, anticorrosive composite coatings embedding engineered mesoporous silica nanoparticles for the protection of AA2024 aluminum substrates were developed. High-surface-area amino-functionalized mesoporous silica nanoparticles were obtained through a high-yield/high-throughput synthesis and, after mild etching, the nanoparticles were functionalized with benzoyl chloride. Through dissociation of the pH-sensitive amide bond, MSN-BC are able to release benzoic acid both in acid and alkaline conditions. The obtained stimuli-responsive nanoparticles were embedded into acrylic coatings applied onto AA2024 alloys in order to test their protective efficiency from corrosion, in comparison to analogue anticorrosive passive coatings.

Results showed that the high surface area of the nanoparticles is accessible and exploitable for the functionalization with benzoyl chloride, allowing to obtain a high grafting of BC. Then, MSN-BC are stable in neutral conditions, and fast release of BA is induced in both acid and alkaline environments. Finally, the results of corrosion tests analyzed through a multitechnique approach based on optical and electron scanning microscopy and electrochemical impedance spectroscopy up to 3 weeks of aging revealed the long-lasting protective efficiency of the smart coatings with respect to passive barrier coatings.

## Supporting Information Available

UV calibration curve for BA; adsorption/desorption isotherm and pore size distribution of MSN; TGA of MSN; EDX spectrum of MSN-NH_2_-E; FTIR spectra of BC and BA; and TGA of MSN-NH_2_-2 (PDF)
