# Supplementary material for: Effectiveness of Mesoporous Silica Nanoparticles Functionalized with Benzoyl Chloride in pH-Responsive Anticorrosion Polymer Coatings
Source: ACS Appl Polym Mater. 2023 Jul 12;5(8):5917–25. doi: 10.1021/acsapm.3c00585 (PMC10426329; doi:10.1021/acsapm.3c00585)
Supplement: Supplementary file 1 — ap3c00585_si_001.pdf [file ap3c00585_si_001.pdf]

Effectiveness of mesoporous silica nanoparticles functionalized with benzoyl chloride in pH responsive anticorrosion polymer coatings

Federico Olivieri[a], Fabio Scherillo[b], Rachele Castaldo[a], Mariacristina Cocca[a], Antonino Squillace[b], Gennaro Gentile§[a], Marino Lavorgna[c]*

[a] Institute of Polymers Composites and Biomaterials, National Research Council of Italy, Via Campi Flegrei, 34, 80078 Pozzuoli (NA), Italy

[b] Department of Chemical, Materials and Industrial Production Engineering, University of Naples Federico II, P.le Tecchio 80, 80125 Naples, Italy

[c] Institute of Polymers Composites and Biomaterials, National Research Council of Italy, P. le Enrico Fermi 1, 80055 Portici (NA), Italy

*Corresponding author. E-mail address: rachele.castaldo@cnr.it

§Corresponding author. E-mail address: gennaro.gentile@cnr.it

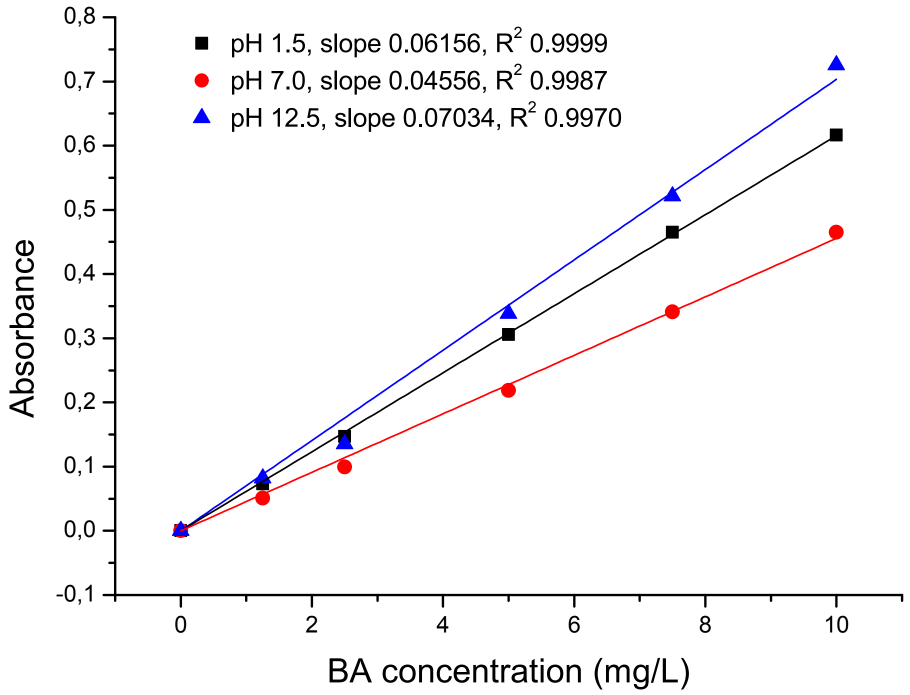

Figure S1. UV calibration curves for BA at different pH values.

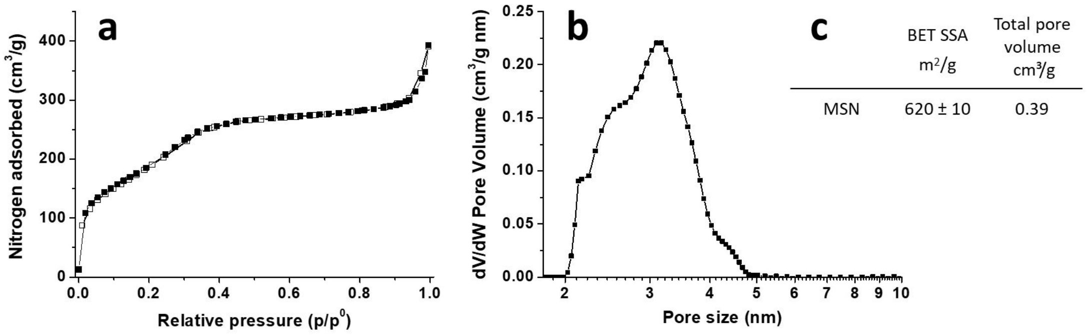

c

	BET SSA m^2/g	Total pore volume cm^3/g
MSN	620 ± 10	0.39

Figure S2. Adsorption/desorption isotherm (a), pore size distribution (b), table of BET SSA and total pore volume (c) of plain MSN.

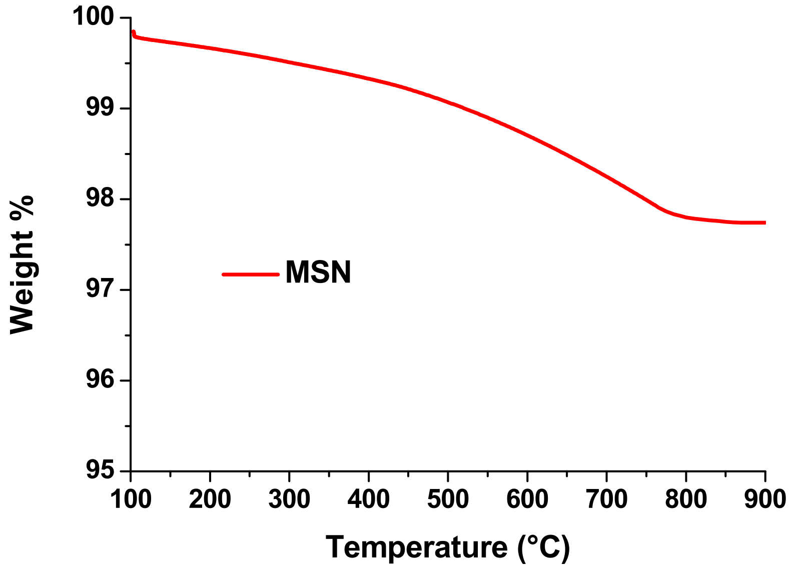

Figure S3. TGA of plain MSN.

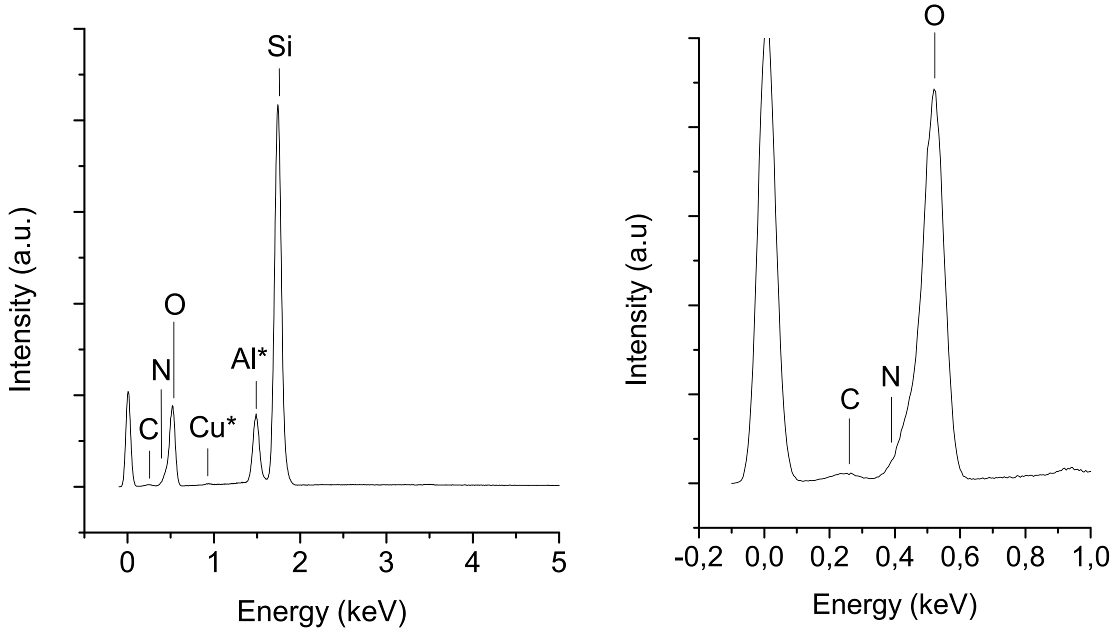

Figure S4. EDX spectrum of MSN-NH_2-E (left, Al and Cu are due to the sample holder) and magnification of the C, N, and O signals (right)

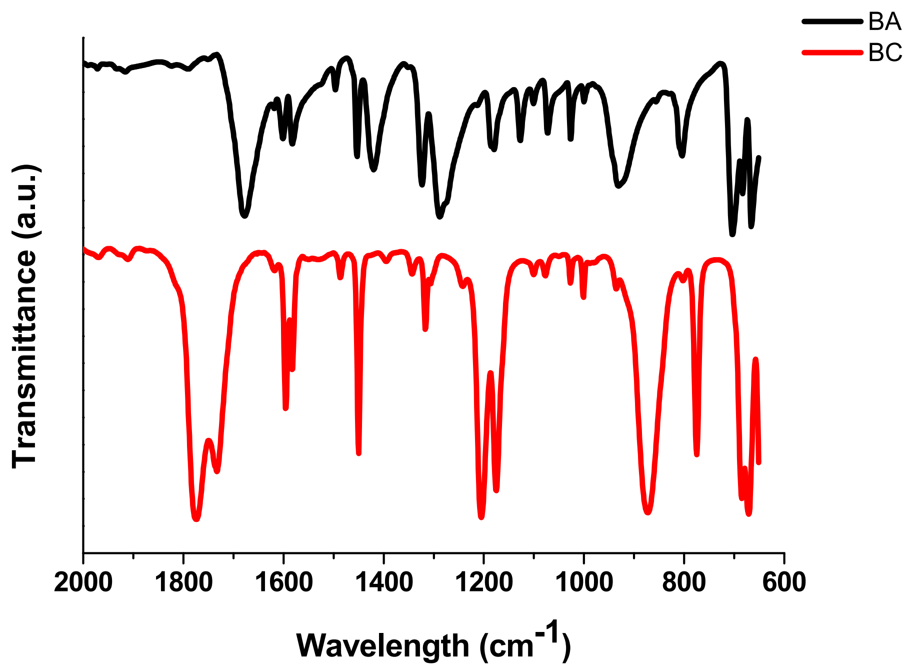

Figure S5. FTIR spectra of benzoyl chloride (BC) and benzoic acid (BA)

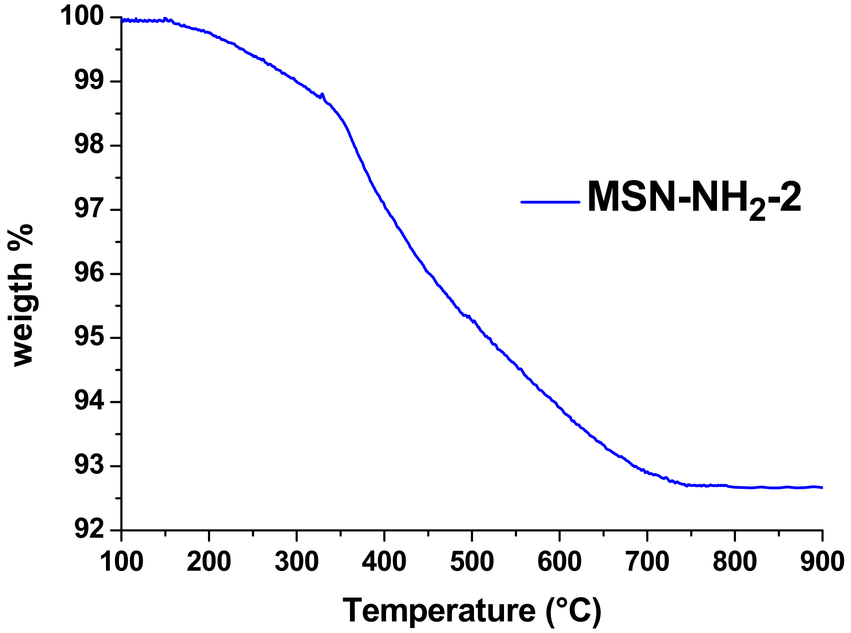

Figure S6. TGA of MSN-NH_2-2 (two steps synthesis).
